# EPS-SJ Exopolisaccharide Produced by the Strain *Lactobacillus paracasei* subsp. *paracasei* BGSJ2-8 Is Involved in Adhesion to Epithelial Intestinal Cells and Decrease on *E. coli* Association to Caco-2 Cells

**DOI:** 10.3389/fmicb.2016.00286

**Published:** 2016-03-09

**Authors:** Milica Živković, Marija S. Miljković, Patricia Ruas-Madiedo, Milica B. Markelić, Katarina Veljović, Maja Tolinački, Svetlana Soković, Aleksandra Korać, Nataša Golić

**Affiliations:** ^1^Laboratory for Molecular Microbiology, Institute of Molecular Genetics and Genetic Engineering, University of BelgradeBelgrade, Serbia; ^2^Instituto de Productos Lácteos de Asturias – Consejo Superior de Investigaciones CientíficasVillaviciosa, Spain; ^3^Center for Electron Microscopy, Faculty of Biology, University of BelgradeBelgrade, Serbia

**Keywords:** *Lactobacillus paracasei*, EPS-SJ, adhesion, *E. coli* association, GALT lymphocytes

## Abstract

The aim of this study was to determine the role of an exopolysaccharide produced by natural dairy isolate *Lactobacillus paracasei* subsp. *paracasei* BGSJ2-8, in the adhesion to intestinal epithelial cells and a decrease in *Escherichia coli*’s association with Caco-2 cells. Annotation of the BGSJ2-8 genome showed the presence of a gene cluster, *epsSJ*, which encodes the biosynthesis of the strain-specific exopolysaccharide EPS-SJ, detected as two fractions (P1 and P2) by size exclusion chromatography (SEC) coupled with multi-angle laser light scattering (MALLS) detection. SEC-MALLS analysis revealed that an EPS-SJ^-^ mutant (EPS7, obtained by insertion mutagenesis of the *glps_2198* gene encoding primary glycosyltransferase) does not produce the P2 fraction of EPS-SJ. Transmission electron microscopy showed that EPS7 mutant has a thinner cell wall compared to the EPS-SJ^+^ strain BGSJ2-83 (a plasmid free-derivative of BGSJ2-8). Interestingly, strain BGSJ2-83 showed higher adhesion to Caco-2 epithelial intestinal cell line than the EPS7 mutant. Accordingly, BGSJ2-83 effectively reduced *E. coli* ATCC25922’s association with Caco-2 cells, while EPS7 did not show statistically significant differences. In addition, the effect of EPS-SJ on the proliferation of lymphocytes in gastrointestinal associated lymphoid tissue (GALT) was tested and the results showed that the reduction of GALT lymphocyte proliferation was higher by BGSJ2-83 than by the mutant. To the best of our knowledge this is the first report indicating that the presence of EPS (EPS-SJ) on the surface of lactobacilli can improve communication between bacteria and intestinal epithelium, implying its possible role in gut colonization.

## Introduction

The components of the cell wall of Gram-positive bacteria may vary considerably depending on the genus, species, and even the strain (Delcour et al., 1999). Hence, the diverse structures of lactobacillus surface molecules, in large part, reflect the adaptation of these microorganisms to specific ecological and environmental conditions.

The surface polysaccharides of lactobacilli contribute significantly to their interaction with the environment, including interactions with other microorganisms and a variety of hosts, in particular with the intestinal mucosa and IEC, contributing to the probiotic characteristics of a specific strain. Bacterial polysaccharides vary in composition, size and the position of branches, contributing to the structural diversity of surface molecules (Lebeer et al., 2008). The polysaccharide component of a bacterial cell’s surface may form EPS that are weakly attached to the cell surface, as well as CPS usually covalently bound to peptidoglycan, forming a firm coating around the cell. It should be noted that the composition, size, and surface properties of polysaccharides depend on the growth conditions of a given bacterial species, as was recently shown for the surface properties of *Lactobacillus acidophilus* NCC2628 (Schär-Zammaretti and Ubbnik, 2003). Based on its chemical composition, an EPS may be a homopolysaccharide – consisting of one type of monosaccharide – or heteropolysaccharide – composed of different types of monosaccharides. Generally, heteropolysaccharides synthesized by lactic acid bacteria may differ in composition, structure, function, size, and molecular weight (de Vuyst and Degeest, 1999). The work of Mozzi et al. (2006) revealed huge diversity of EPS produced by 31 strains belonging to different species of lactic acid bacteria. The molecular weight of the EPS varied between 8 to >5,000 kDa. Interestingly, it was shown that low molecular weight-EPS are more common than high molecular weight-EPS in lactic acid bacteria (Mozzi et al., 2006). For instance, it has been shown that the EPS of *L. casei* YIT 9018 is composed of two types of heteropolysaccharides (Nagaoka et al., 1990).

Generally, the EPS biodiversity could be result of different sugar nucleotide biosynthesis pathways and genetic variability of the strains (Mozzi et al., 2006). The genetic elements involved in the synthesis of the conserved structure of the polymer are organized into operons, located on chromosomal or plasmid DNA (Boels et al., 2001; Zivkovic et al., 2015). The genes present in the *eps* operons encode proteins with specific functions: assembling of repeating units, chain length determination, polymerization, export, and regulation (de Vuyst and Degeest, 1999). A number of glycosyltransferases involved in the repeating units’ assembly have been revealed (Mozzi et al., 2006). Characterization of a large number of operons for the biosynthesis of surface polysaccharides has revealed that within the conserved structural organization, variations in the number and position of genes in the operon exist (Péant et al., 2005).

Due to their long history of safe use in human consumption, lactobacilli have been given “Qualified Presumption of Safety” status (EFSA, 2007). They have been proposed as beneficial for human and animal health as probiotics, defined as “live microorganisms which when administered in adequate amounts confer a health benefit on the host” (FAO-WHO, 2006).

The literature data suggest that surface polysaccharides indirectly affect the adhesive properties of lactobacilli by means of various interactions such as passive forces, electrostatic interactions, and hydrophobic and steric forces (Servin, 2004). The work of Nikolic et al. (2012) showed that EPS-producing strain *L. paraplantarum* BGCG11 have lower adhesion to IEC than its non-EPS-producing derivative NB1. The results of Ruas-Madiedo et al. (2006) showed that the adhesion levels depend on the EPS type. For example, the EPS fraction GG showed no effect on the adhesion of *L. rhamnosus* GG, while at the highest dose it slightly increased the adherence of *Bifidobacterium animalis* IPLA-R1. However, the EPS fractions NB667 and IPLA-R1 promoted a significant reduction in the adhesion of both probiotic strains *B. longum* NB667 and *B. animalis* IPLA-R1. Further, Denou et al. (2008) showed that deletion of the entire EPS cluster slightly increased the gut persistence time of the knock-out mutant in comparison to the parental strain. In addition, the results of Polak-Berecka et al. (2014) indicated that polysaccharides produced by *L. rhamnosus* E/N hindered adhesion and aggregation by masking protein receptors at the cell surface. Moreover, the reduction in EPS production by *L. johnsonii* FI9785 increased bacterial adhesion to chicken gut explants (Dertli et al., 2015).

Lactobacillus strains *L. paraplantarum* BGCG11 and *L. fermentum* BGHI14 have been shown to have immunomodulatory capacity and to have the ability to induce the production of various cytokines (IL-17, IFN-γ, IL-10, TNF-α, and IL-1β; Nikolic et al., 2012; Lukic et al., 2013). The EPS-producing strain *L. paraplantarum* BGCG11, as an example, was shown to exhibit an anti-inflammatory and immunosuppressive profile, whereas its non-EPS derivative induced a pro-inflammatory response (Nikolic et al., 2012).

Interest in the health-promoting potential of lactobacilli has significantly increased, especially with regard to their role in the prevention and treatment of disease. Our previous results showed that the natural dairy isolate *L. paracasei* subsp. *paracasei* BGSJ2-8 synthesizes BacSJ bacteriocin, PI-type proteinase and AggLb aggregation promoting protein, and it is able to survive in simulated gut conditions, hence it could be used in dairy industry as functional starter culture with potential probiotic traits (Lozo et al., 2007; Miljkovic et al., 2015). In this study, the effects of an EPS produced by BGSJ2-8 (EPS-SJ) on its adhesion to IEC, a decrease of *Escherichia coli* association with Caco-2 cells, and the proliferation of lymphocytes in GALT were investigated. The knowledge obtained in this study may help to understand the specific contribution of EPS-SJ in gut colonization and pathogen exclusion.

## Materials and Methods

### Media and Growth Conditions

The strains and plasmids used in this study are listed in **Table 1.** Lactobacillus strains were grown in De Man-Rogosa-Sharpe (MRS) medium (Merck GmbH, Darmstadt, Germany) at 28°C, 30°C or 37°C when it was appropriate. *E. coli* strains, used as intermediate hosts for cloning experiments and plasmid propagation, were grown in LBwith aeration at 37°C for 16 h. Solid media were prepared by adding agar (1.5% w/v). When appropriate, the media contained ampicillin (100 μg/ml) or erythromycin (250 μg/ml for *E. coli*, or 2.5 μg/ml for lactobacilli).

**Table 1:** List of strains, plasmids and primers used in this study.

Strain	Relevant characteristics	Reference
*Lactobacillus paracasei* subsp. *paracasei*		
BGSJ2-8	BacSJ^+^, Agg^+^, EPS-SJ^+^	Laboratory collection
BGSJ2-83	Plasmid free derivative of the strain BGSJ2-8, BacSJ^-^, Agg^-^, EPS-SJ^+^	Kojic et al., 2010
BGCG11	EPS-CG11^+^	Nikolic et al., 2012
BGHN14	Agg^-^	Kojic et al., 1991
BGSJ2-83-2198-EPS7 (EPS7)	Derivative of the strain BGSJ2-83, EPS-SJ^-^	This study
*Escherichia coli*		
DH5α	F^-^, Δ*lac, U169(Φ80 lacZ*Δ*M15), supE44, hsdR17, recA1, gyrA96, endA1, thi–1, relA1*	Hanahan, 1983
EC101	*supE thi* (*lacproAB*) (F9 *traD36 proAB lacI*q *Z*DM15), *repA* from pWV01	Law et al., 1995
ATCC25922	Serotype O6, Biotype 1	FDA strain Seattle 1946

**Plasmids and constructs**	**Relevant characteristics**	**Reference**

pA13	Em^r^, *lacZ*, plasmid pA1 derivative, 3.9 kb	Kojic et al., 2010
pGhost9	Em^r^, Ts, 4.6 kb	Maguin et al., 1996
pGhost9/2198	Plasmid pGhost9 derivative, carrying PCR fragment with part of the *glps_2198* gene complementary to chromosomal DNA of BGSJ2-8	This study
pGEM-T Easy Vector	3015 bp, Amp^r^, bacterial transient vector with high level expression	Promega

**Primers (target)**	**Primer sequence 5′–3′**	**Reference**

2198 Fw (*glps_2198*)	CGGGGACTGACGCCAGC	This study
2198 Rv (*glps_2198*)	GCAACCATCTCTGAAAATCCGAGG
2198a	CAGCATCTGAGCACAGAACAG
pGhF (pGhost plasmid)	GGGGGATGTGCTGCAAGGCG	This study
pGhR (pGhost plasmid)	GTCCGTTAAATCGACTGGCG


*BacSJ^-^, absence of bacteriocin BacSJ production; Agg^-^, absence of autoaggregation phenotype; EPS-SJ^+^, producer of EPS-SJ; EPS-SJ^-^, absence of EPS-SJ production; Em^*r*^, resistance to erythromycin; Amp^*r*^, resistance to ampicillin.*

### Sequencing and Annotation of the Putative EPS Biosynthetic Cluster

Genome sequencing was performed by Macrogen (Seoul, Korea), a next generation sequencing service. The nucleotide sequence of the gene cluster *glps_2198-2211* (*epsSJ*) was deposited in the “European Nucleotide Archive” database^1^ (Accession No. LN879393). An in-depth analysis of this gene cluster was performed. The nucleotide sequence of each gene was used for BLAST searches against the NCBI nucleotide database. When the best BLASTN hit had less than 50% coverage, a BLASTP analysis was performed.

### DNA Manipulation and Construction of EPS^-^ Mutants

Chromosomal DNA from BGSJ2-8 was isolated as described by Hopwood et al. (1985). For cloning, part of the *glps_2198* gene was amplified by PCR using primers 2198 Fw and 2198 Rv complementary to the *glps_2198* gene of BGSJ2-8 (**Table 1**). PCR amplification was performed using a KAPA *Taq* DNA polymerase kit (Kapa Biosystems, Wilmington, MA, USA, 5 U/ml) and amplified in Gene Amp PCR system 2700 (Applied Biosystems, Waltham, MA, USA) using the following program: predenaturation (7 min, 94°C), 30 successive cycles consisting of denaturation (30 s, 94°C), annealing (1 min, 50°C), elongation (30 s, 72°C), and a final elongation (7 min, 72°C). The resulting PCR amplicon, 2198 Fw/2198 Rv, was purified using QIAquick PCR Purification KIT/250 (QIAGEN GmbH, Hilden, Germany), sequenced [Macrogen, Europe (Amsterdam, The Netherlands)], and ligated into pGEM-T Easy cloning vector (Promega, Fitchburg, WI, USA). General procedures for cloning and DNA manipulation were performed as described by Sambrook et al. (1989). The ligation mixtures were used to transform *E. coli* DH5α by a standard heat-shock transformation procedure (Sambrook et al., 1989). Transformants were selected on LB plates containing ampicillin (100 μg/ml). The resulting pGEM-T Easy/2198 was sequenced. Using the BLAST algorithm^2^ for nucleotide sequence analysis, it was confirmed that fragment 2198 cloned into pGEM-T Easy/2198 was identical (100%) to the corresponding sequences in the genome of BGSJ2-8. The construct pGEM-T Easy/2198 was further digested by *Eco*RI (Fermentas, Vilnius, Lithuania). The obtained 2198 fragment (518 bp) was eluted using a QIAquick Gel Extraction Kit (Qiagen, Hilden, Germany), and ligated into pGhost9 plasmid digested by *Eco*RI restriction enzyme. The ligation mixture was used to transform *E. coli* EC101. Transformants were selected on LB plates containing erythromycin (250 μg/ml). The resulting construct, pGhost9/2198, carrying the appropriate fragment, was used for transformation of strain BGSJ2-83 by electroporation (Holo and Nes, 1995) using an Eppendorf electroporator (Eppendorf, Hamburg, Germany). The BGSJ2-83/pGhost9/2198 transformants were selected on MRS plates containing erythromycin (5 μg/ml) at 28°C, and the presence of plasmid pGhost9/2198 was confirmed.

All plasmid isolations from *E. coli* were performed using the QIAprep Spin Miniprep kit according to the manufacturer’s instructions (Qiagen, Hilden, Germany). Plasmids from *L. paracasei* were isolated using the same kit with the previously described modification for *L. lactis* (Filipic et al., 2013). Transposition was performed as described by Maguin et al. (1996). A logarithmic culture of BGSJ2-83/pGhost9/2198 was grown at 28°C (a permissive temperature for the replication of plasmid pGhost9), and then transferred to 37°C for 48 h, to allow the integration of the plasmid into the host chromosome. The potential EPS^-^ mutants were screened on MRS plates containing erythromycin (5 μg/ml) as erythromycin-resistant colonies, after 48 h at 37°C. The integration of plasmid pGhost9/2198 into the *glps_2198* gene was confirmed by PCR using primers complementary to pGhost9 (pGhF and pGhR) and the 5′ of the *glps_2198* gene, upstream of the sequence used for the insertion mutagenesis (**Table 1**). PCR was performed essentially as described above with an annealing temperature of 55°C. Finally, the integration of plasmid pGhost9/2198 into the mutant’s chromosome was specifically analyzed by Southern hybridization of the chromosomal DNA of strain BGSJ2-83 and its EPS^-^ derivative BGSJ2-83-2198-EPS7 (in further text EPS7) digested by *Eco*RI or *Hin*dIII by using PCR fragment 2198 as a probe. Southern blot hybridization and detection of positive signals was done on the membrane, as recommended by the manufacturer (Roche Diagnostic GmbH, Mannheim, Germany).

### EPS-SJ Extraction and Purification

EPS-SJ extraction and purification from strains BGSJ2-83 (*epsSJ*^+^), EPS7 (the *epsSJ* knock-out) and BGHN14 (*epsSJ*^-^) was performed essentially as described by Nikolic et al. (2012). Bacterial strains were grown at 25°C for 48 h in a basal minimum medium (Cerning et al., 1994) inoculated with an overnight culture grown in basal minimum medium (10%, v/v) and incubated. After incubation, the bacteria were removed by centrifugation (12,000 × *g*, 30 min, 4°C) and EPS was extracted and precipitated at 4°C for 48 h by adding two volumes of chilled absolute ethanol. The precipitate was collected by centrifugation (12,000 × *g*, 20 min, 4°C), dissolved in distilled water and dialyzed against water, using 12–14 kDa MWCO membranes (Sigma Chemical Co., St. Louis, MO, USA), for 24 h at 4°C. The dialyzed retentate was freeze-dried to obtain the EPS-crude fraction which was additionally purified to reduce the DNA and protein content as previously described (Nikolic et al., 2012). The resulting lyophilized powder was the purified EPS, which was used in different concentrations for further analyses. The protein content of this purified EPS was quantified by the BCA method (Pierce, Rockford, IL, USA), the presence of DNA was determined using the a Take3 Multi-volume Plate (Biotek Instruments GmbH, Bad Friedchshall, Germany) and the EPS content was determined by means of SEC coupled with MALLS analysis (SEC-MALLS).

### Size Exclusion Chromatography

The molar mass distribution of the purified EPS was analyzed by means of a SEC-MALLS detector (Dawn Heleos II; Wyatt Europe GmbH, Dembach, Germany) as previously described (Nikolic et al., 2012). Briefly, the chromatographic system used (Waters, Milford, MA, USA) has a photodiode array detector, set at 280 nm for detecting proteins, and a 410 refractive index detector for quantifying the different peaks obtained after separation (40°C, 0.45 ml/min, 0.1 M NaNO_3_) in the TSKgel G3000PWXL and TSKgel G5000 PWXL columns, placed in series (Supelco–Sigma, Bellefonte, PA, USA). Quantification of the EPS-fractions was made from the refractive index signal using the regression equations obtained from Dextran standards of different molar mass. The molar mass distribution of the EPS-fractions was obtained using Astra version 3.5 software (Wyatt Europe GmbH, Dembach, Germany).

### Transmission Electron Microscopy

The samples of bacterial colonies were encased in Bacto-agar, and the solidified blocks were cut into 1 mm^3^ cubes and fixed with 2.5% glutaraldehyde (v/v) in 0.1 M Sørensen PBS. After postfixation with 2% osmium teroxide in the same buffer, samples were dehydrated using increasing concentrations of ethanol and embedded in Araldite resin (Fluka, Neu-Ulm, Germany). Ultra-thin sections were obtained using a Leica UC6 ultramicrotome (Leica Microsystems, Mannheim, Germany) and mounted on copper grids. For the ultrastructural analysis of the cell wall’s structural components, serial sections were counterstained with 5% phosphotungstic acid in distilled water for 30 min at 56°C and subsequently with 0.1% Alcian blue in 3% acetic acid for 30 min at room temperature. Sections were examined on a Philips CM12 transmission electron microscope (Philips/FEI, Eindhoven, The Netherlands) equipped with a SIS MegaView III digital camera (Olympus Soft Imaging Solutions, Hamburg, Germany). The digital images were used for both cell wall ultrastructural analysis and the cell wall thickness measurements. The cell wall thickness was measured by Image J software (NIH, Bethesda, MD, USA) at a magnification of 53,000×. About 50 randomly selected sections of whole bacterial cells were used for the measurements at five different regions of each cell.

### Adhesion to Caco-2 and HT29-MTX Cells

The colonocyte-like cell lines Caco-2 and HT29-MTX were used to determine the adhesion ability of the lactobacillus strains to mucosal surfaces in the intestine. A Caco-2 cell line was purchased from the European Collection of Cell Cultures (ECACC No. 86010202) and HT29-MTX was kindly supplied by Dr. T. Lesuffleur (INSERM UMR S 938, Paris, France; Lesuffleur et al., 1990). The culture and maintenance of the cell lines were carried out as previously described (Nikolic et al., 2012) using Advanced DMEM medium (Gibco Invitrogen, Paisley, UK) for Caco-2 and HT29-MTX supplemented with heat inactivated fetal bovine serum (5% for Caco-2, 10% for HT29-MTX), L-glutamine (2 mM) and with a mixture of antibiotics (10 U/ml penicillin, 10 μg/ml streptomycin, 50 μg/ml gentamicin). Media and reagents were purchased from PAA (Pasching, Austria). Intestinal cells (passage 5–6) were seeded in 24-well plates and cultivated until a confluent differentiated state was reached (monolayer). Lactobacillus strains were cultured for 24 h and, after washing twice with Dulbecco’s PBS solution (Sigma, St. Louis, MO, USA), were resuspended in the corresponding cell-line media without antibiotics at a concentration of about 10^8^ CFU/ml. The cellular monolayers were also carefully washed and lactobacillus suspensions were added at a ratio of about 10:1 (lactobacilli:eukaryotic cells). The eukaryotic cells were counted in Tripan blue solution. Adhesion experiments were carried out for 1 h at 37°C, 5% CO_2_ and afterward the wells were gently washed to release unattached bacteria before proceeding with the lysis of cellular monolayers using 0.25% Trypsin–EDTA solution (PAA, Pasching, Austria). Dilutions of samples, before and after adhesion, were made in PBS solution and lactobacilli counts were performed in MRS agar plates. The adhesion was calculated as a percentage: CFU adhered lactobacilli strain/CFU added lactobacilli strain. Experiments were carried out in two replicated plates and in each plate two wells were used per sample.

### Decrease on *E. coli* Association to Caco-2 by Lactobacilli

The capability of reference strain *E. coli* ATCC25922 to be associated to the intestinal epithelium in the presence and absence of lactobacilli was tested. Bacterial cultures were washed twice with PBS and resuspended in DMEM without antibiotics at a concentration of ∼1 × 10^8^ CFU/ml; this number was corroborated by plate counting in the agar medium specific for each bacterium. The bacterial suspensions containing *E. coli* ATCC25922 or a combination of *E. coli* and lactobacilli (ratio 1:1) were independently added to the Caco-2 monolayers at a ratio of 10:1, (bacteria:eukaryotic cells; in the case of *E. coli*–lactobacilli combinations, each bacterial type was added at a ratio of 5:1) and incubated at 37°C, with 5% CO_2_ for 1 h. Afterward, the monolayers were gently washed twice with PBS to remove the unattached bacteria, and the eukaryotic cells were released using 0.25% Trypsin–EDTA solution (Sigma, St. Louis, MO, USA). The samples were diluted in 0.9% (w/v) NaCl buffer and plated to LA to enumerate the associated *E. coli*. The percentage of *E. coli* association was calculated as follows: 100 × CFU/ml bacteria associated CFU/ml bacteria added (the dilution of bacteria was taken into account). Each combination was tested in triplicate. To determine the capability of the lactobacilli to decrease the association of *E. coli* ATCC25922 to Caco-2 monolayers, data were referred to that obtained with the *E. coli* alone (i.e., 100% association). Each combination of *E. coli*–lactobacilli, and the *E. coli* alone as reference, were tested in three replicates.

### Proliferation of GALT-Isolated Lymphocytes in the Presence of Non-Viable Lactobacilli

The lactobacilli cells were inactivated by UV light for three cycles of 30 min each. Plate counting was carried out after UV treatment to corroborate the absence of live lactobacilli that might be able to recover in the proper medium. UV-inactivated lactobacilli were then divided in single use aliquots, frozen in liquid N_2_ and stored at –80°C until use (López et al., 2012). This study was approved by the Animal Experimentation Ethical Committee of the Faculty of Pharmacy, University of Belgrade (Serbia), in strict adherence to international directives. A total number of three Wistar rats (healthy female adults between 6 and 8 weeks old) were purchased from the Farm of the Military Medical Academy, Belgrade (Serbia). For the experiments each animal was anesthetized with CO_2_ and, once assured of the loss of corneal reflex, its intestine was excised from the jejunum to the ileocaecal junction. The whole small intestine was placed in cold Hank’s balanced salt solution (HBSS without calcium and magnesium ions, prepared according to the formulation of Gibco, Invitrogen, Carlsbad, CA, USA) and kept at 4°C until processing. Finally, the animals were sacrificed using the increase of CO_2_ concentration. The isolation of lymphocytes from GALT (Peyer’s Patches lymphocytes and intestinal epithelium lymphocytes) was carried out as previously described (Hidalgo-Cantabrana et al., 2014a).

To quantify the response of GALT to the different factors tested, 2 × 10^5^ lymphocyte cells were incubated with UV-inactivated lactobacilli (at a ratio of 1:5) for 4 days in complete RPMI medium with antibiotics at 37°C and 5% CO_2_. All cultures were performed in triplicate in 96-well round-bottom microtiter plates. After 4 days of incubation, the proliferation of GALT-lymphocytes was determined with a Cell Proliferation Assay Kit (Millipore Corporation, Billerica, MA, USA) following the manufacturer’s instructions. Results were compared to a negative control (lymphocytes growing in complete RPMI medium with antibiotics).

### Statistical Analysis

After checking the normal distribution of the proliferation data, one-way ANOVA tests were used to determine the differences between each factor and the negative control. Finally, one-way ANOVA tests together with the mean comparison test of least significant difference were used to compare the differences between the three strains. The results are represented as mean ± standard deviation or standard error. The SPSS 15.0 statistical software package (SPSS Inc, Chicago, IL, USA) was used for all determinations and *p* < 0.05 was considered significant. The Kruskal–Wallis test (Non-parametric ANOVA) followed by Dunn’s multiple comparison test were used to compare the mean values obtained at transmission electron microscopy. Significant differences were set at α = 0.05.

## Results

### Identification of a *glps_2198-2211* (*epsSJ*) Gene Cluster

The entire genome of *L. paracasei* subsp. *paracasei* BGSJ2-8 was sequenced, annotated and partly assembled (unpublished, in house data). Although BGSJ2-8 does not produce visible ropy EPS, genome annotation revealed the presence of a unique chromosomally located gene cluster *glps_2198-2211* (*epsSJ*) encoding for strain-specific EPS-SJ synthesis (**Table 2**). Most of the 14 genes in the cluster showed similarities to various known proteins involved in the biosynthesis of extracellular or CPS from other lactic acid bacteria, including genes for enzymes responsible for regulation, polymerization, export, assembling repeating units, and chain length determination (Hidalgo-Cantabrana et al., 2014b). The *epsSJ* operon starts with the genes *glps_2211*, *glps_2210*, and *glps_2209* that could be putatively involved in spatial and temporal regulation of EPS-SJ synthesis and in chain length determination. The *glps_2198* gene, encoding a priming glycosyltransferase, is positioned at the end of the *epsSJ* gene cluster. The priming glycosyltransferase has a role in the first (priming) step in the EPS biosynthesis. Upstream of the *glps_2198* there are four genes (*glps_2199*, *glps_2204*, *glps_2206*, and *glps_2207*) assigned as glycosyltransferases with function of transferring the sugars of the EPS subunit in an ordered way. The gene *glps_2200*, as a putative galactoside acetyltransferase, might have be a factor modifying glycosyl residue, while the *glps_2202*, with homology to *CpsIbJ*, a putative β1, 3-galactosyltransferase, might have a role in the addition of galactose to the EPS-SJ polymer.

**Table 2:** *L. paracasei* BGSJ2-8 strain-specific EPS cluster.

Gene ID	Best BlastN/BlastP^∗^ hits	Identities	*E*-value	Coverage
*glps_2198*	*Lactobacillus casei* Shirota *cps1J* (AB470649), priming glycosyltransferase	498/672 (74%)	6.00*E* - 110	100%
*glps_2199*	EpsV protein *[Lactobacillus paracasei* subsp. *paracasei* 8700:2]#	156/315 (49%)#	8*e* - 85#	98%#
*glps_2200*	*Lactobacillus casei* Shirota *cps1G* (AB470649), galactoside acetyltransferase	412/574 (71%)	3.00*E* - 75	85%
*glps_2201*	*Streptococcus thermophilus* LY03 *eps* gene cluster;	131/187 (70%)	4.00*E* - 12	18%
	membrane protein [*Oenococcus oeni* ATCC BAA-1163]#	*137/314#*	*1e* - *69*#	*67%#*
*glps_2202*	*CpsIbJ* [*Streptococcus agalactiae*]#	81/229 (35%)#	5e - 39#	73%#
*glps_2203*	*EpsL* [*Streptococcus thermophilus*]#	95/315 (30%)#	2e - 35#	99%#
*glps_2204*	glycosyltransferase [*Lactobacillus paracasei* subsp. *paracasei* 8700:2]#	120/385 (31%)#	8e - 39#	98%#
*glps_2205*	predicted protein [*Lactobacillus paracasei* subsp. *paracasei* 8700:2]#	97/364 (26%)#	2e - 15#	92%#
*glps_2206*	*Oenococcus oeni* PSU-1, glycosyltransferase	255/395 (64%)	2.00*E* - 11	46%
*glps_2207*	*Leuconostoc citreum* KM20, glycosyltransferase	670/992 (67%)	2*e* - 81	81%
*glps_2208*	*Plasmodium falciparum* 3D7	23/23 (100%)	0.58	13%
*glps_2209*	*Lactobacillus rhamnosus* RW-6541M, Wze (AY659978);	558/741 (75%)	3.00*E* - 139	99%
	*Lactobacillus casei* Shirota *cps1B* (AB470649), chain length determination	523/690 (75%)	2.00*E* - 135	92%
*glps_2210*	*Lactobacillus rhamnosus* RW-6541M, *wzd* (AY659978)	265/382 (69%)	5.00*E* - 33	81%
*glps_2211*	*Lactobacillus rhamnosus* GG, chain length regulator	258/349 (73%)	1.00*E* - 52	84%
	*Lactobacillus casei* Shirota *cps1A* (AB470649), chain length determination	247/345 (71%)	3.00*E* - 41	84%


*^∗^When the best BLASTN hit has less than 50% coverage, a BLASTP analysis was performed. Best BlastP hit is shown in the table; #The scores of BlastP results.*

**FIGURE 1:**
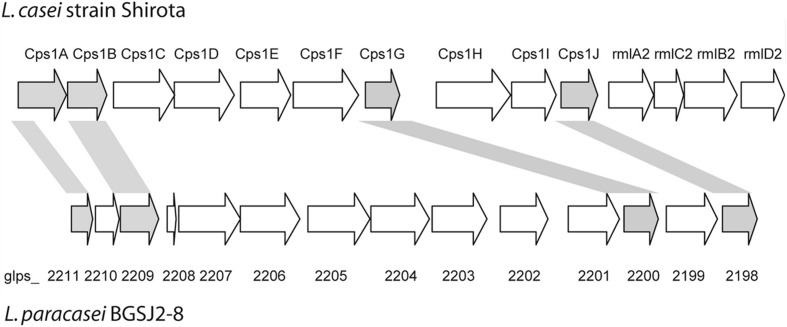
**Schematic representation of CPS and EPS-SJ synthesis clusters in *L. casei* Shirota and *L. paracasei* BGSJ2-8, respectively.** Genes sharing high sequence similarity are indicated and highlighted in the same colors. Adapted from Yasuda et al. (2008).

Interestingly, several genes in the *epsSJ* gene cluster have the best BLAST hits to the gene cluster for biosynthesis of the CPS previously described in probiotic *L. casei* Shirota (CPS operon Shirota; Yasuda et al., 2008) (**Figure 1**). The nucleotide sequences of genes *glps_2198* (518 bp), *glps_2200* (435 bp), *glps_2209* (524 bp), and *glps_2211* (269 bp) of strain BGSJ2-8 showed identity to the nucleotide sequences of the genes *cps1J* (74%), encoding primary glycosyltransferases, *cps1G* (71%), encoding galactoside acetyltransferase, and *cps1B* (75%) and *cps1A* (71%), encoding polysaccharide chain length determination proteins, within the Shirota CPS operon.

### Construction and Analysis of the EPS-SJ^-^ Mutants

In order to determine the functional properties of the annotated *epsSJ* gene cluster, the gene encoding the primary glycosyltransferase *glps_2198* was disrupted by insertional inactivation as described in Section “Materials and Methods.” Initially, the isolation of plasmid DNA from the erythromycin resistant colonies obtained after the transposition experiment indicated that the construct pGhost9/2198 had been integrated into the chromosome of BGSJ2-83. The experimental work was continued with a strain designated as EPS7, a potential mutant in the *glps_2198* gene encoding the primary glycosyltransferase. EPS7 showed a similar growth phenotype to the parental strain (data not shown). The chromosomal integration of plasmid pGhost9/2198 was initially verified by isolation of plasmid DNA from the EPS7 mutant and transformation of competent *E. coli* EC101. After 24 h of incubation at 37°C, transformants were not obtained, indicating the absence of circular forms of plasmid pGhost9/2198 in the EPS7 mutant strain. Additionally, the integration was confirmed by PCR and Southern blot hybridization (as described in section “Materials and Methods”). The results revealed that plasmid pGhost9/2198 had been integrated into the chromosomal DNA of EPS7 at the specific site of the *glps_2198* gene (data not shown).

### Molar Mass Distribution of EPS

SEC-MALLS analysis was performed to analyze the molar mass distribution of the purified polymeric material and to quantify the EPS fractions. The strain *L. paracasei* subsp. *paracasei* BGHN14 was used as a negative reference strain, since PCR analysis confirmed that the *epsSJ* genes are not present in this strain (data not shown).

**FIGURE 2:**
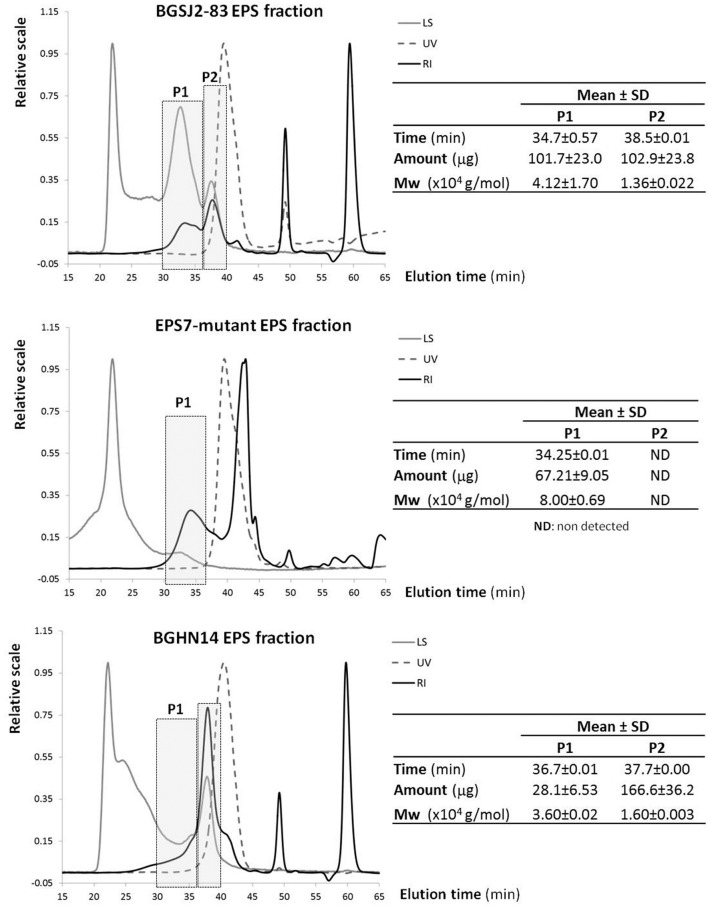
**SEC-MALLS of the EPS fractions purified from *L. paracasei* subsp. *paracasei* BGSJ2-83 and from strain BGHN14, lacking the *epsSJ* gene cluster.** LS, light scattering detector at 90^o^ angle; UV, photodiode array detector set at 280 nm; RI, refraction index detector; Mw, weight/molar mass.

The results showed that the polymer purified from strain BGSJ2-83 presents two EPS fractions synthesized in similar amounts (**Figure 2**). One has a higher molecular weight (P1), about 41,200 Da, whereas the second fraction is smaller, about 13,600 Da (P2). However, it is worth mentioning that the P2 fraction might also contain proteins, since in the elution time corresponding to this peak (around 38 min) there was also an increase in the signal corresponding to the photodiode array detector in the UV spectrum (280 nm). Interestingly, in the EPS purified from EPS7 (the mutant), in which the primary glycosyltransferase *glps_2198* gene was disrupted by insertional inactivation, the P2 fraction was not detected (although the increase in the UV signal is present), while the P1 fraction is present in lower amount (2/3 of amount detected in BGSJ2-83) and with higher molecular weight (about 80,000 Da). In the case of EPS purified from strain BGHN14, lacking the entire *epsSJ* gene cluster, the highest production corresponded to the fraction coinciding with the P2 EPS-SJ (1.6 higher amount than in BGSJ2-83), also presenting the same increase in the UV signal. Indeed, the amount of protein quantified by BCA was similar in both EPS fractions, i.e., 15.6 ± 0.5 μg/ml and 15.1 ± 0.3 μg/ml for BGSJ2-83 and BGHN14, respectively.

### Electron-Microscopic Analysis and Cell Wall Thickness Measurement

**FIGURE 3:**
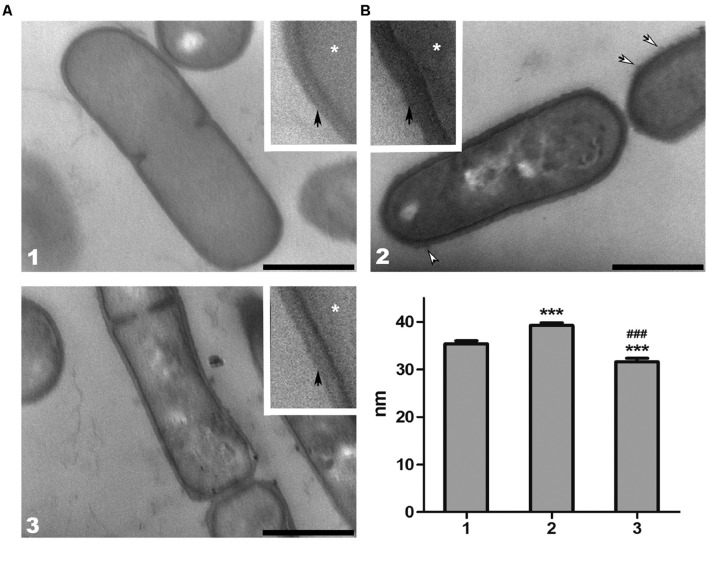
**(A)** Representative transmission electron micrographs of thin sections of *L. paracasei* subsp. *paracasei* (1) BGHN14, (2) BGSJ2-83, and (3) EPS7. The samples were prepared for ultrastructural analysis and ultra-thin sections were counterstained with phosphotungstic acid/Alcian blue. The details of the cell walls were enlarged (insets). White arrows indicate the fibrillar polysaccharide protrusions of the cell wall, while asterisks indicate cytoplasm. The thin, electron-dense layer at the surface of the cell wall suggests the presence of an S-layer (black arrows) in all analyzed strains. Scale bars: 500 nm. **(B)** Cell wall thickness of *L. paracasei* subsp. *paracasei* (1) BGHN14, (2) BGSJ2-83, and (3) EPS7 strains. Values are presented as means ± standard error of the mean. Statistically significant differences vs. non-EPS-SJ producing BGHN14: ^∗∗∗^*p* < 0.001; statistically significant differences vs. BGSJ2-83: ^###^*p* < 0.001.

Both, electron-microscopic analysis (**Figure 3A**), and cell wall thickness measurement (**Figure 3B**) of *L. paracasei* subsp. *paracasei* strains, showed a significant decrease of cell wall thickness in EPS7 and BGHN14 strains compared to BGSJ2-83 (∼40 nm). Indeed, it was observed that EPS7, lacking the P2 EPS fraction (∼30 nm), had even thinner cell wall than BGHN14 lacking P1 (∼35 nm).

In addition, ultrastructural analysis of the different strains of *L. paracasei* subsp. *paracasei* demonstrated an underlying alteration in cell wall structure. In BGHN14 and EPS7, the cell walls appeared pale, with low electron-density, particularly in the EPS7 mutant. The outer surface of the cell wall of these groups was smooth, without visible protrusions. Quite the opposite, the cell wall of BGSJ2-83 had a dark, electron-dense appearance. Also, the cell wall of BGSJ2-83 was slightly curved in shape with additional protrusions of fibrillar polysaccharide material at the extracellular surface.

Furthermore, detailed examination revealed the presence of a thin, electron-dense layer at the outermost surface of the cell wall in all of the groups, suggesting the presence of proteins, particularly in EPS7 and BGHN14, where this layer was clearly visible. In contrast, the presence of proteins in BGSJ2-83 was harder to resolve, and in most cases it was visibly absent.

### Characterization of the Role of EPS-SJ in Adhesion to Epithelial Intestinal Cells

**FIGURE 4:**
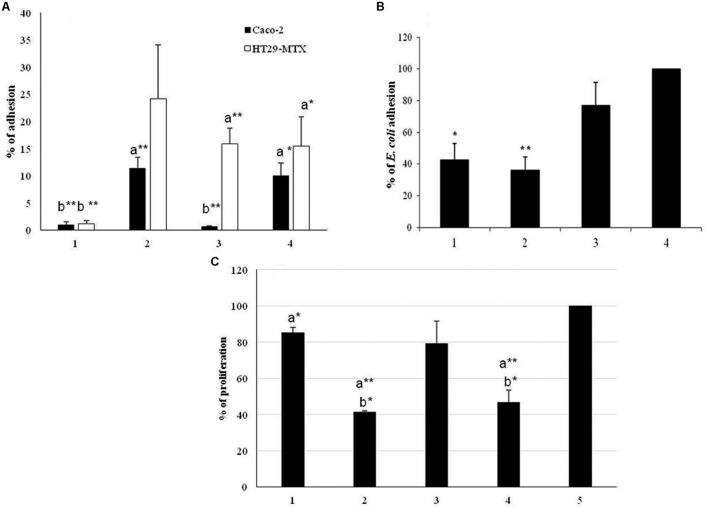
**(A)** Adhesion of the strain *L. paracasei* subsp. *paracasei* BGSJ2-83 and the EPS-SJ^-^ mutant EPS7 to the human IEC lines Caco-2 (black bars) and HT29-MTX (white bars). 1. BGCG11; 2. BGSJ2-83; 3. EPS7; 4. BGHN14. The statistical differences between each strain with respect to the control strains are annotated with asterisks (^∗^*p* < 0.05, ^∗∗^*p* < 0.001) and letters (a – BGCG11 and b – BGHN14). **(B)** Association of *E. coli* to Caco-2 cells in the presence of *L. paracasei* subsp. *paracasei* BGSJ2-83, EPS7 and *L. paraplantarum* BGCG11. 1. *E. coli*/BGCG11; 2. *E. coli*/BGSJ2-83; 3. *E. coli*/EPS7; 4. *E. coli*. The statistical differences between each strain with respect to *E. coli* are annotated with asterisks (^∗^*p* < 0.05, ^∗∗^*p* < 0.001). **(C)** Proliferation of GALT cells in the presence of *L. paracasei* subsp. *paracasei* BGSJ2-83 and EPS7. 1. BGCG11; 2. BGSJ2-83; 3. EPS7; 4. BGHN14; 5. L cells. The statistical differences between each strain with respect to the control lymphocytes and control strain BGCG11 are annotated with asterisks (^∗^*p* < 0.05, ^∗∗^*p* < 0.001) and letters (a – GALT, b – BGCG11).

To estimate the role of EPS-SJ in the adhesion ability of strain BGSJ2-8 to IEC, *in vitro* comparative analysis of strain BGSJ2-83 and its EPS-SJ^-^ derivative EPS7 was performed. The strains BGCG11, producing high molecular weight EPS-CG11, and BGHN14, lacking *epsSJ* gene cluster, were used as controls. In particular, the EPS-SJ-producing strain BGSJ2-83 and strain BGHN14 showed higher percentages of adhesion to the Caco-2 cell line than the mutant, EPS7 (**Figure 4A**).

In parallel, the adhesion ability of strains BGSJ2-83 and EPS7 was tested on the HT29-MTX cell line, characterized by mucus production, which is the first barrier to the interaction of bacteria with IEC (Lesuffleur et al., 1990). Again, BGSJ2-83 showed a higher percentage of adhesion to HT29-MTX cells than EPS7, although in this case BGHN14 showed low adhesion ability, similar to EPS7 (**Figure 4A**).

### Characterization of the Role of EPS-SJ in Decrease on *E. coli* Association to Caco-2 Cells

In this study, we analyzed the potential of EPS-SJ-producing and non-producing strains to compete with *E. coli* and to reduce the association capability of *E. coli* to Caco-2 epithelial cells (**Figure 4B**). The *E. coli* strain ATCC25922 was significantly less associated to Caco-2 cells in the presence of BGSJ2-83 and control strain BGCG11 than when it was analyzed alone, indicating the ability of all lactobacilli strains to diminish the association of *E. coli* to Caco-2 cells. *E. coli* exhibited the lowest association rate to the Caco-2 cell line when co-incubated with the strain BGSJ2-83, which produces EPS-SJ, as well as in the presence of control strain BGCG11. No significant differences were detected between the two strains. In contrast, when *E. coli* was co-incubated with EPS7 the association of *E. coli* to Caco-2 cells was reduced, but not to the same extent as in the presence of the EPS producing strains.

### Effect of Insertional Mutagenesis on the Role of EPS-SJ on Proliferation of GALT-Isolated Lymphocytes

In order to monitor the effect of EPS-SJ’s presence on the cell surface of BGSJ2-83, the proliferation of the primary GALT culture in the presence of UV-irradiated cells of BGSJ2-83 and EPS7 was followed during 3 days of co-incubation. The strains BGCG11, producing high molecular weight EPS-CG11, and BGHN14, lacking *epsSJ*, were used as reference strains. The results are expressed as a percentage relative to the cells incubated in RPMI medium alone, with a standard error (**Figure 4C**). The results show that UV-irradiated cells of both BGSJ2-83 and EPS7 induced lower levels of lymphocyte proliferation compared to the control GALT-isolated lymphocytes. Interestingly, the level of proliferation of GALT-isolated lymphocytes determined for the EPS7 mutant was higher compared to BGSJ2-83, indicating that EPS-SJ P1, present on the cell surface of BGSJ2-83 and absent on the cell surface of EPS7, contributed to the lower proliferation of GALT-isolated lymphocytes.

## Discussion

In this study, we have investigated the role of exopolysaccharide EPS-SJ, produced by the natural dairy isolate *L. paracasei* subsp. *paracasei* BGSJ2-8, in adhesion to IEC, decreased association of *E. coli* to Caco-2 cells and proliferation of GALT-isolated lymphocytes. Our results show that the unique *epsSJ* gene cluster identified in the chromosome of *L. paracasei* subsp. *paracasei* BGSJ2-8 is involved in the biosynthesis of strain-specific EPS-SJ, containing two EPS fractions, P1 and P2. Yasuda et al. (2008) remarked that *L. casei* Shirota produced EPS with two peaks of different molecular weight, named polysaccharide-peptidoglycan (PS-PG) polymer. In addition, Yasuda et al. (2008) showed that the mutation of different genes in the cluster from *cps1A* to *cps1J* modified the synthesis of high molecular weight-EPS (named PS1), but a smaller fraction (PS2) remained intact.

In our study, the results of a SEC-MALLS analysis revealed that insertional mutagenesis of the *glps_2198* gene, encoding priming glycosyltransferase, led to the absence of the P2 fraction in EPS7, related to low molecular weight EPS-SJ (13,600 Da) present in BGSJ2-83, as well as to an increase in molecular weight of the P1 and its reduced production. According to literature data the gene encoding priming glycosyltransferase is highly evolutionarily conserved (Jolly and Stingele, 2001) and could be located at the beginning of the *eps* operons, as reported for *L. helveticus* NCC2745 (Jolly et al., 2002) and *L. paraplantarum* BGCG11 (Zivkovic et al., 2015), in the middle of the *eps* clusters, as in *Streptococcus thermophilus* strains (Stingele et al., 1996; Almiron-Roig et al., 2000), *L. lactis* NIZO B40 (Kleerebezem et al., 1999), and *L. delbrueckii* subsp. *bulgaricus* Lfi5 (Lamothe et al., 2002), or in the vicinity of the end of the *eps* clusters as in the case of *L. rhamnosus* (Péant et al., 2005) and here in the case of BGSJ2-8. The priming glycosyltransferase is involved in the first step of biosynthesis of an oligosaccharide “block.” It is a member of the EPS biosynthesis polyprenyl glycosyl phosphotransferases family with the role of transferring the sugar from UDP-glucose or UDP-galactose to a lipid carrier. The results obtained in this study revealed that interruption of the *glps_2198* gene completely affected the biosynthesis of the low molecular weight P2 fraction of EPS-SJ, rather than the high molecular weight P1 fraction, although the P1 fraction is synthesized in lower amount. The results are in accordance to work of Mozzi et al. (2006) where it was reported that low molecular weight EPS are more common in lactic acid bacteria than high molecular weight. Hence, it could be deduced that the gene *glps_2198* encoding priming glycosyltransferase in BGSJ2-83 is necessary for the first steps in biosynthesis of EPS-SJ.

Increased electron-density as a consequence of specific polysaccharide phosphotungstic acid/Alcian blue counterstaining suggests a significant ratio of EPS in the cell wall of this strain. These fibrillar protrusions probably correspond to EPS-SJ, the presence of which was subsequently confirmed by SEC-MALLS analysis, detected in BGSJ2-83.

The results from electron-microscopic analysis and cell wall measurement clearly indicate that both fractions of EPS-SJ, present on the cell surface of BGSJ2-83, contribute to the thickness of the cell wall in this strain. In fact, the absence of the P1 and P2 fractions in BGHN14 and EPS7, respectively, influenced the decrease of cell wall thickness by approximately 5 and 10 nm. Besides its lower content, this could also be a consequence of increased electron-density of the peptidoglycan layer due to increased polysaccharide content.

Probiotic lactobacilli exert their positive effect on human health by various mechanisms. The adhesive properties of probiotic strains provide several important abilities: the colonization of intestinal mucosa, persistence in the intestine, the competitive exclusion of pathogenic microorganisms, and immunomodulation (Muñoz-Provencio et al., 2009). In fact, the colonization of intestinal mucosa is an important criterion proposed by FAO/WHO guidelines for the selection of strains with probiotic potential, since their health-promoting effects might be partly dependent on their persistence in the intestine and adhesion to mucosal surfaces (Collado et al., 2009). Hence, the influence of EPS-SJ on the potential to adhere to the intestinal mucosal surface was examined using widely accepted *in vitro* models, Caco-2 and HT29-MTX cells.

It has been suggested that the adhesive properties of lactobacilli largely depend on the surface properties of probiotic strains (e.g., EPS), enabling them to interact with the mucosa and IEC (Segers and Lebeer, 2014). It can be assumed that the better adhesion of BGSJ2-83 and BGHN14 than the EPS7 mutant to Caco-2 cells is probably due to the presence of EPS-SJ P2 on the cell surface of strains BGSJ2-83 and BGHN14, which ensures better communication between bacteria and IEC. In contrast, the strain BGCG11, producing ropy EPS-CG11, was previously described to have low adhesion ability, which was correlated with the presence of EPS-CG11 on the cell surface (Nikolic et al., 2012).

It has been shown previously that the adhesion of bacteria to IEC is better in the absence of cell surface polysaccharides, due to better exposure of cell surface proteins in bacteria to the adhesin in eukaryotic cells (Ruas-Madiedo et al., 2006; Denou et al., 2008; Nikolic et al., 2012; Polak-Berecka et al., 2014; Dertli et al., 2015). Interestingly, EPS-SJ P2 is most likely positively involved in the adhesion of the BGSJ2-8 strain to IEC. Specifically, our results revealed that the presence of the EPS-SJ P2 around the bacteria increases adhesion to Caco-2 cells, in contrast to previously published data. Particularly, Nikolic et al. (2012) showed that presence of EPS-CG11 negatively affected adhesion of *L. paraplantarum* BGCG11 to IEC. In addition, Polak-Berecka et al. (2014) suggested that polysaccharides produced by *L. rhamnosus* E/N interfere with adhesion by masking protein receptors at the cell surface. However, the results of Ruas-Madiedo et al. (2006) demonstrated that the adhesion of bacteria to IEC depends on the EPS type and the dose. Such, the EPS GG produced by *L. rhamnosus* GG at the highest dose increased the adherence of *Bifidobacterium animalis* IPLA-R1. Probably it could be referred to differences in physicochemical and/or structural characteristics of the EPS polymers, as well as to the surface characteristics of the strains that contribute to the differences in adhesion (Ruas-Madiedo et al., 2006). Hence, it is likely that the molecular structure of EPS-SJ P2 matrix contributes to its adhesion to IEC, although the role of EPS molecules in the adhesion of bacteria to IEC still remains unclear.

Due to the fact that the presence of mucin in the HT29-MTX cell line simulates the bacterial interaction with gut mucus (McGuckin et al., 2011), the adhesion to HT29-MTX cells was also tested. In general, the tested strains showed a higher percentage of adhesion to the HT29-MTX cell line, while their adhesion ability to the Caco-2 cell line was lower. Similarly, the adhesion levels of strains *L. rhamnosus* DR20, *L. acidophilus* HN017, and *Bifidobacterium lactis* DR10 were two to three times higher to HT29-MTX cells compared to the degree of adhesion to Caco-2 cells (Gopal et al., 2001). These findings are in contrast to our previous results obtained with other putative probiotic strains, where the adhesion to Caco-2 cells was significantly higher than to HT29-MTX cells (Uroić et al., 2014). It is possible that the presence of the glycoprotein layer (mucin) on the surface of HT29-MTX cells participates in the interaction with the cell surface compounds of bacterial cells, which contribute to their hydrophobicity. Furthermore, the adhesion of strain BGHN14 and the EPS7 mutant, lacking the P1 and P2, respectively, was lower than the adhesion of BGSJ2-83 to HT29-MTX cells. However, there is no significant difference between the adhesion of BGHN14 and EPS7 strains to HT29-MTX, indicating that EPS-SJ P2 more specifically contributes to the adhesion of BGSJ2-83 and EPS7 to IEC than to the gut mucus matrix.

The other important health promoting property of probiotic strains is their capability to neutralize the negative effects of pathogens (FAO-WHO, 2006). This characteristic is shown to be strain-dependent and several mechanisms of action have been proposed to explain it: (i) a physical blocking of the pathogen’s entry (colonization competition), (ii) induction of mucus production, (iii) reinforcement of the selective permeability of the epithelium by increasing tight-junctions, (iv) production of antimicrobial factors, and, among others, (v) stimulation of the innate immune response (Gareau et al., 2010; Liévin-Le Moal and Servin, 2014). In this study we have shown that EPS-SJ-producing strain BGSJ2-83 exhibits higher adhesion to Caco-2 cells and is more capable of competing for the colonization of the intestinal niche than *E. coli* strain ATCC25922. Similarly, non-EPS producing derivative of EPS-CG11 (NB1) more efficiently reduced the association of *Clostridium difficile* or *Yersinia enterocolitica* than parental BGCG11 strain (Zivkovic et al., 2015). Interestingly, the presence of EPS-CG11-producing strain BGCG11, exhibiting low adhesion to Caco-2 cells, decreased the association of *E. coli* ATCC25922 to Caco-2 cells, similarly to previously obtained results by Zivkovic et al. (2015). Hence, it could be concluded that both EPS-CG11 and EPS-SJ are effective macromolecules protecting the IEC from pathogen binding regardless of their adhesion ability (Zivkovic et al., 2015). It is most likely that *E. coli* cells failed to access the IEC surface because they were trapped within the EPS matrix. It has been previously reported that different lactobacillus species have the ability to reduce the association of diverse enteropathogens to intestinal cells (García-Cayuela et al., 2014). This capability has also been attributed to antimicrobial activity related to the production of bacteriocins or organic acids (Satish-Kumar et al., 2011; Kaewnopparat et al., 2013). Since strain BGSJ2-83 is a BGSJ2-8 derivative lacking the plasmid carrying the genes responsible for bacteriocin production and does not exhibit antimicrobial activity, it is more likely that its capability is related to the presence of specific structural components on the cell surface. It has been proposed that the cell surface components involved in this feature could be cell surface associated proteins (Varma et al., 2010), S-layer macromolecules built from proteins (Zhang et al., 2010) and aggregation factors (Kaewnopparat et al., 2013), as well as EPS (Zivkovic et al., 2015). Hence, the results obtained in this study indicate that EPS-SJ could be involved in the decreased association of *E. coli* to Caco-2 cells. Since the results of this study show that association of *E. coli* with Caco-2 cells is higher in the presence of EPS7 than in the presence of BGSJ2-83, it can be concluded that EPS-SJ P2, lacking in EPS7, specifically contributes to this feature.

Finally, an important feature of potential probiotic candidates is the capacity to modulate the host’s immune response either in the terms of strengthening the immunological barrier against pathogen, or decreasing the immune responsiveness in exaggerated inflammatory conditions. The study on *L. casei* Shirota’s *cps* gene cluster has shown that this operon determines the synthesis of a specific polysaccharide moiety relevant for immune regulation (Yasuda et al., 2008). The production of high molecular weight PS1 was directly related to the suppressive effect of this strain upon IL-6 production in macrophages stimulated with LPS (Yasuda et al., 2008). The results of our study indicate that the presence of EPS-SJ on the cell surface contributes to the lower level of GALT-isolated lymphocyte proliferation. The level of GALT-isolated lymphocyte proliferation stimulated with EPS7 mutant was higher compared to BGSJ2-83, indicating that EPS-SJ P2, present on the cell surface of BGSJ2-83, has an inhibitory effect on GALT-isolated lymphocyte proliferation. The GALT is intestinal frontier and essential component of systemic immune response, which protects body from pathogens and foreign antigens. On the other hand it enables tolerance to commensal bacteria and dietary antigens and protects mucosa from detrimental inflammatory immune responses (Spahn and Kucharzik, 2004; Jung et al., 2010). Hence, GALT has a pivotal role in the decision between immune response and tolerance. The lower level of GALT-isolated lymphocytes proliferation in the presence of EPS-SJ-producing strain, BGSJ2-83, could indicate modulation of the immune response toward the tolerogenic environment. This feature could be useful in treatment of excessive inflammatory condition such as Crohn’s disease and other inflammatory disorders which occur as a result of an inappropriate innate and/or adaptive immune response. However, further experiments are needed in order to decipher the molecular mechanism of this feature.

## Conclusion

The results obtained in this study indicate that EPS-SJ, specifically the P2 fraction, interacts with a certain host cell component, providing better gut colonization and pathogen exclusion. These results may contribute to the previous knowledge of the role of EPS in adhesion to IEC. Namely, it was established previously that polysaccharides hindered bacterial adhesion to host by masking protein receptors involved in the adherence to IEC. Here, we report that EPS may positively or negatively affect adherence of bacteria to the host cells.

## Author Contributions

MŽ – conception and design of the work, performed main work, analyzed, interpreted and critically revised the data; MSM and MT – construction and characterization of the knock-out, draft of the work related to molecular genetics; PR-M – conception and design of SEC-MALLS, performed the SEC-MALLS analyses, draft related to SEC-MALLS and critically revised the manuscript; MBM – performed work and made part of the draft related to transmission electron microscopy; KV and MT – performed a work related to adhesion, *E. coli* association to Caco-2 cells, and GALT proliferation, made part of the draft and critically revised the manuscript; SS – EPS characterization; AK – conception and design, performed the work related to transmission electron microscopy, made the draft and critically revised the manuscript; NG – conception and the design of the work, supervised the complete work, analyzed and interpreted the data, draft the work, critically revised the manuscript. All authors finally approved the version to be published and agreed to be accountable for all aspects of the work in ensuring that questions related to the accuracy or integrity of any part of the work are appropriately investigated and resolved.

## Conflict of Interest Statement

The authors declare that the research was conducted in the absence of any commercial or financial relationships that could be construed as a potential conflict of interest.

## Footnotes

1 http://www.ebi.ac.uk/ena

2 http://www.ncbi.nlm.nih.gov/BLAST

## Abbreviations

CFU: colony forming units
CPS: capsular polysaccharides
EPS: extracellular polysaccharides
IEC: intestinal epithelial cells
GALT: gastrointestinal associated lymphoid tissue
LB: Luria Broth
MALLS: multi-angle laser light scattering
MRS: De Man-Rogosa-Sharpe medium
PBS: phosphate buffer solution
SEC: size exclusion chromatography
